# Upgrading the definition of early gastric cancer: better staging means more appropriate treatment

**DOI:** 10.7497/j.issn.2095-3941.2015.0054

**Published:** 2015-12

**Authors:** Luca Saragoni

**Affiliations:** Department of Pathology, G.B. Morgagni-L. Pierantoni Hospital, Forlì 47121, Italy

**Keywords:** Early gastric cancer (EGC), definition, diagnosis, prognosis, treatment

## Abstract

Since Murakami defined early gastric cancer (EGC) as a “carcinoma limited to the gastric mucosa and/or submucosa regardless of the lymph node status”, several authors have focused on the most influential histopathological parameters for predicting the development of lymph node metastases by considering the lymph node status as an important prognostic factor. A few authors have also considered the depth of invasion as one of the keys to explaining the existence of subgroups of patients affected by EGC with poor prognoses. In any case, EGC is still considered an initial phase of tumor progression with good prognosis. The introduction of modern endoscopic devices has allowed a precise diagnosis of early lesions, which can lead to improved definitions of tumors that can be radically treated with endoscopic mucosal resection or endoscopic submucosal dissection (ESD). Given the widespread use of these techniques, the Japanese Gastric Cancer Association (JGCA) identified in 2011 the standard criteria that should exclude the presence of lymph node metastases. At that time, EGCs with nodal involvement should have been asserted as no longer fitting the definition of an early tumor. Some authors have also demonstrated that the morphological growth pattern of a tumor, according to Kodama’s classification, is one of the most important prognostic factors, thereby suggesting the need to report it in histopathological drafts. Notwithstanding the acquired knowledge regarding the clinical behavior of EGC, Murakami’s definition is still being used. This definition needs to be upgraded according to the modern staging of the disease so that the appropriate treatment would be selected.

## Introduction

Early gastric cancer (EGC) is commonly reported in Japan^1^^,^^2^ because of the widespread use of organized screening programs. However, the number of EGC cases is apparently rising in other parts of the world^3^^-^^6^. At the same time, minimally invasive treatments of EGC have been emerging. In particular, therapeutic endoscopic submucosal dissection (ESD) has been introduced as a treatment option^7^^,^^8^. Although excellent results have been reported, concerns have been expressed about the increased rates of local recurrence and early cancer-related deaths after therapeutic endoscopy^9^^-^^11^. Patients selected for ESD should have no lymph node metastases and involved cut ends^12^^,^^13^. However, the current definition of EGC does not consider lymph node status^14^^-^^16^. This review aims to provide an upgraded definition of EGC in the era of minimally invasive treatments, including endoscopy, by critically examining the data from the literature focusing on tumor-specific factors associated with the risk of lymph node metastases.

## EGC: challenges

The term EGC, defined in 1971 by the Japanese Society of Gastroenterology and Endoscopy^17^ as a carcinoma limited to the mucosa and/or submucosa regardless of the lymph node status, has continued to trigger a controversy over the years^18^^-^^20^. Numerous studies have focused on the key parameters that can be associated with the risk of lymph node metastases or treatment failure in EGC^21^^-^^25^. The major problem in microstaging is represented by the lack of agreement regarding the characteristics that truly classify nonthreatening EGCs, which have excellent prognosis with survival rates of 98%-100%, compared with threatening tumors that have an increased incidence of lymph node metastases (15%-20%) and survival rates of approximately 70%. Over the past years, the most important factor has seemed to be the depth of invasion. In 1991, Inoue *et al.*^26^ reported that the 5-year survival rate is 100% in patients with mucosal lesions and 90% in those with submucosal invasion. They also demonstrated that lymph node metastases exerted a major effect on prognoses ranging from an overall survival rate of 99%, if N_0_, to 73%, if N_1_. In the following years, other authors demonstrated that the tumors infiltrating the submucosa of a stomach are associated with a significant increase in the incidence of lymph node metastases^8^^,^^27^^-^^30^ and a poor prognosis^31^. In particular, mucosal tumors have a 6% mean incidence of lymph node metastases versus 28% of submucosal EGCs. However, the depth of infiltration into the wall is not the only prognostic factor and/or predictive parameter of the increased risk of lymph node metastases. In fact, the morphological growth patterns of the lesions should be assessed by pathologists because of their important prognostic meaning. Since Kodama described the morphological growth patterns of EGC in 1983^32^ by combining them with the size of the lesions, only few Western authors have considered Kodama’s classification (**Table 1**)^32^, thereby showing its important prognostic meaning^6^^,^^23^^-^^25^. In particular, we have demonstrated in our previous studies that lymph node metastases and Kodama’s PEN A types are the only independent negative prognostic factors in EGCs. In our experience, Kodama’s PEN A type has an incidence of lymph node metastases of 31.7% and a death risk nearly four times higher (hazard ratio =3.91) than those of all the other Kodama types. Thus, we suggested using Kodama’s classification in ESD and surgical specimens to advise an appropriate treatment to clinicians. When a lymph node status was considered by itself, a few authors determined that patients with EGC who have three or more positive lymph nodes have a significantly poor prognosis^33^^-^^35^. In our series, we showed that patients with EGC who have more than three positive nodes have a significantly poor prognosis similar to that of patients with advanced disease with a death risk approximately 13 times higher than that of patients with negative lymph nodes (hazard ratio =12.78), as demonstrated by the multivariable analysis^6^. If lymph node metastases are present, patients must be treated surgically by adding chemotherapy in the adjuvant setting according to the recommendations of the European Society of Medical Oncology^36^, given the obvious benefits of adjuvant therapy in these patients^37^^-^^42^. Other important risk factors for lymph node metastases are the presence of lymphovascular invasion and tumor dedifferentiation^5^^,^^9^^,^^27^^-^^29^^,^^33^^,^^35^^,^^43^^-^^54^. Moreover, histologic diffuse type of Lauren and size larger than 2 cm are significantly linked with a higher incidence of lymph node involvement^55^^-^^77^.

**Table 1:** Kodama’s classification

Kodama’s types	Description
Small mucosal	
Mucosal (M)	Intramucosal EGCs measuring less than 4 cm
Submucosal (SM)	Intramucosal EGCs minimally invading submucosa measuring less than 4 cm
Super mucosal	
Mucosal (M)	Intramucosal EGCs measuring more than 4 cm
Submucosal (SM)	Intramucosal EGCs minimally invading submucosa measuring more than 4 cm
Pen (penetrating)	
A	EGCs massively invading submucosa with nodular pattern measuring less than 4 cm
B	EGCs massively invading submucosa with saw teeth pattern measuring less than 4 cm
Mixed	Penetrating types (A or B) measuring more than 4 cm

EGC, early gastric cancer.

In particular, the mean incidence of lymph node metastases is 9% in the absence of lymphovascular invasion *vs.* 53% in the opposite situation. Well-differentiated tumors have a lymph node involvement in 13% of the cases *vs.* 34% of poorly differentiated ones, whereas tumors smaller than or equal to 2 cm present nodal metastases in 8% of the patients *vs.* 25% of those larger than 2 cm.

According to the same literature data, the likelihood of lymph node metastases is 7.1, 6.2, and 3.8 times more probable if the tumor infiltrates the submucosa (*vs.* mucosa), if a lymphovascular invasion exists (*vs.* not), and if it was Lauren’s diffuse type (*vs.* intestinal type), respectively.

According to the macroscopic types (**Table 2**) defined by Paris classification^78^, the probability of lymph node metastases is 2.3 times higher for depressed lesions (*vs.* elevated EGCs).

**Table 2:** Macroscopic classification of EGC

Macroscopic types	Description
Type 0 I	Protruded
Type 0 IIa	Elevated
Type 0 IIb	Flat
Type 0 IIc	Depressed
Type 0 III	Excavated

EGC, early gastric cancer.

Some authors also determined other important predictive factors of lymph node metastases when lymph node involvement is considered, and/or the significant role of a few parameters previously mentioned in predicting nodal positivity is reinforced. In particular, Fukuhara *et al.*^79^ noted that both lymphovascular invasion and young age are significant predictive factors of lymph node metastases in EGCs for non-Asian ethnic groups. Yang *et al.*^80^ determined deep submucosal invasion, antral location, and venous invasion as the predictive parameters of lymph node involvement. Gotoda *et al.*^44^ showed that the incidence of nodal metastases increases (from 9% to 24%) as the depth of tumor infiltration increases within the submucosal layer. Shida *et al.*^81^ demonstrated that lymphatic and venous invasions are independent predictive factors of lymph node metastases.

Considering all these data, some attempts exist to update the definition of EGC and to determine the risk factors associated with the likelihood of lymph node metastases by improving the diagnostic ability. Based on the current definition, the risk of lymph node metastases in patients with EGC is 15%-24%^2^^,^^71^^,^^82^. This finding cannot be ignored, and the case for upgrading the definition of EGC should be based on the exclusion of lymph node involvement. This observation is particularly true in an era of endoscopic treatment for “fitting” patients with EGC.

The indications for endoscopic treatment of EGC have undergone changes while the pioneering techniques of the Japanese were evolving^30^^,^^43^^,^^44^^,^^83^^-^^85^. When the National Cancer Center Hospital of Tokyo (Japan) first introduced the indications for mucosal endoscopic mucosal resection in 1987^86^, the lesions had to be smaller than 15 mm. Current indications for therapeutic endoscopic resection are referred to ESD according to the Japanese Gastric Cancer Association (JGCA)^12^, and the following “standard” criteria should be fulfilled:

- Intramucosal tumor;
- Well-differentiated intestinal tumor type according to Lauren;
- Tumor size <2 cm;
- Absence of neoplastic ulcer;
- Absence of lymphovascular invasion;
- Negative horizontal and deep margins.

If all these parameters are satisfied, the ESD diagnostic procedure can be also considered therapeutic.

JGCA has recently provided the “expanded” indications for ESD^12^ for investigative purposes. These expanded indications^43^^,^^44^ include tumors T_1a_ (mucosal only) exhibiting the following:

- Differentiated type, ulcerative findings (ULs) (−), but >2 cm in diameter;
- Differentiated type, ULs (+) and not more than 3 cm in diameter;
- Undifferentiated type, ULs (−) and not more than 2 cm in diameter.

The “standard” criteria are strongly recommended because they are considered safe because of the decreased risk of lymph node metastases. This consideration translates into the presence of lymph node metastases as an absolute contraindication to therapeutic endoscopy. Hence, the current definition of EGC, which does not consider the lymph node status, must be upgraded. However, a standardized upgraded definition of EGC shared by both Western and Eastern pathologists cannot be easily found.

First, we should try to standardize the diagnosis of EGC. In fact, Japanese pathologists base their diagnoses of EGC only on cytological atypia and architectural distortion, whereas their Western colleagues define EGCs as tumors that invade the proper lamina. The classification of Vienna^87^ apparently solved the problem. However, after Stolte^88^ performed the revision, the differences could not be bypassed. The 7th edition of the classification by the World Health Organization (WHO)^89^, shared by Western and Eastern pathologists, defined only invasive tumors as EGCs. Low- and high-grade intraepithelial lesions were also considered depending on cytological atypia as noninvasive counterparts. In addition, according to WHO, the criteria of invasiveness are sometimes not easily defined. They are also apparently poorly reproducible, depending on a subjective evaluation.

Some Western and Eastern authors showed that some lesions defined as high-grade dysplasia by Western pathologists hid submucosal EGCs in ESD specimens^90^.

Nevertheless, we should ask ourselves how to define EGC without considering this topic. Should we simply call “early” those tumors that do not have lymph node metastases or other things are needed? In our previous series of 530 patients with EGC^6^, we provided new elements for an updated definition of EGC. In particular, we suggested calling “early” those gastric cancers that are limited to the mucosa, or at least, invade minimally the submucosa, lesions without potential risk of developing lymph node metastases. This study is a retrospective monocentric investigation with a long follow-up and standardized surgery and histological diagnosis, which showed that tumors with a size of more than 2 cm (66.7%), infiltration of the submucosa (74.0%), diffuse histological type (29.2%), and Kodama’s PEN A type (42.3%) are important and significant risk factors for lymph node metastases. An agreement must be determined on what we mean by and how to measure the minimal invasion of the submucosa. According to the classification of Paris^78^, we should define those EGCs infiltrating the submucosal layer with a maximum depth of 500 micron (SM1 tumors) and minimally invading the submucosa. According to Kodama’s classification, we should not use quantitative criteria. However, only minimal invasion of the submucosa should be considered an initial infiltration under the muscularis mucosae. According to our previous data, these lesions have the same incidence of lymph node metastases and similar 5- and 10-year survival probability as that of mucosal tumors. In conclusion, we suggest regarding the lesions that are limited to the mucosa or those that invade minimally the submucosa according to Kodama’s criteria and the lesions without lymph node metastases as EGCs. The size of tumors should be also considered, given the significant increased risk of lymph node involvement for lesions larger than 2 cm.
